# The associations of long-term physical activity in adulthood with later biological ageing and all-cause mortality – a prospective twin study

**DOI:** 10.1007/s10654-024-01200-x

**Published:** 2025-01-17

**Authors:** Anna Kankaanpää, Asko Tolvanen, Laura Joensuu, Katja Waller, Aino Heikkinen, Jaakko Kaprio, Miina Ollikainen, Elina Sillanpää

**Affiliations:** 1 Gerontology Research Center (GEREC), Faculty of Sport and Health Sciences, University of Jyväskylä, Jyväskylä, Finland; 2 Methodology Center for Human Sciences, University of Jyväskylä, Jyväskylä, Finland; 3 Faculty of Sport and Health Sciences, University of Jyväskylä, Jyväskylä, Finland; 4 Institute for Molecular Medicine Finland (FIMM), HiLife, University of Helsinki, Helsinki, Finland; 5 Minerva Foundation Institute for Medical Research, Helsinki, Finland; 6 Wellbeing services county of Central Finland, Jyväskylä, Finland

**Keywords:** DNA methylation, Mortality, Lifespan, Biological age, Physical activity

## Abstract

Objectives: The association between leisure-time physical activity (LTPA) and a lower risk of mortality is susceptible to bias from multiple sources. We investigated the potential of biological ageing to mediate the association between long-term LTPA and mortality and whether the methods used to account for reverse causality affect the interpretation of this association. Methods: Study participants were twins from the older Finnish Twin Cohort (*n* = 22,750; 18–50 years at baseline). LTPA was assessed using questionnaires in 1975, 1981 and 1990. The mortality follow-up lasted until 2020 and biological ageing was assessed using epigenetic clocks in a subsample (*n* = 1,153) with blood samples taken during the follow-up. Using latent profile analysis, we identified classes with distinct longitudinal LTPA patterns and studied differences in biological ageing between these classes. We employed survival models to examine differences in total, short-term and long-term all-cause mortality, and multilevel models for twin data to control for familial factors. Results: We identified four classes of long-term LTPA: sedentary, moderately active, active and highly active. Although biological ageing was accelerated in sedentary and highly active classes, after adjusting for other lifestyle-related factors, the associations mainly attenuated. Physically active classes had a maximum 7% lower risk of total mortality over the sedentary class, but this association was consistent only in the short term. After accounting for familial factors and excluding participants reporting prevalent cardiovascular diseases, LTPA exhibited less favourable associations with mortality. Conclusion: The association between LTPA and lower all-cause mortality may be largely due to genetic confounding and reverse causality.

**Supplementary Information:**

The online version contains supplementary material available at 10.1007/s10654-024-01200-x.

## Introduction

The association between leisure-time physical activity (LTPA) and a lower risk of mortality from all causes and cardiovascular diseases is frequently reported [1–5]. However, the evidence is generally based on observational studies, and LTPA is typically assessed at single time points. Studies using repeatedly measured LTPA have suggested that being both persistently and increasingly active over adulthood are associated with a reduced risk of mortality [2]. However, evidence based on randomized controlled trials (RCTs) has failed to confirm that LTPA prevents premature mortality [6]. This may be due to the lack of exercise trials designed with mortality as a primary outcome, carried out with high long-term compliance and with sufficient statistical power to detect the effect [7, 8], but also to the absence of causal relationship between LTPA and mortality.

Genetically informed studies have suggested that genetic selection may partly account for the association between LTPA and mortality, as genetically healthy participants tend to engage in LTPA [9, 10]. In fact, the association is susceptible to reverse-causality bias: an underlying suboptimal physiological or predisease state may negatively affect LTPA, which means that the observed association may be due to a causal relationship between the covert disease state and subsequent premature death [11]. Previous studies have shown that increased control for reverse causality results in weaker associations between LTPA and mortality [11, 12]. Moreover, researchers have proposed that, rather than LTPA per se reducing mortality risk, participation in LTPA and the ability to increase LTPA in later life are themselves indicators of good fitness and health [8]. Although there is no concluding evidence on causal relationship between LTPA and lower mortality, there are numerous widely recognized health benefits of LTPA which have been supported not only by RCTs but also by genetically informed studies. These include, for example, improvements in tissue health (e.g. adipose tissue, heart, arteries, bones and brain) [13–15] and physical fitness [16].

Slower biological ageing is a plausible mechanism for explaining the path from an active lifestyle (or a healthy phenotype) to reduced mortality risk. Biological ageing is the gradual and progressive decline in system integrity that occurs with advancing age and results in increased risk of morbidity and mortality [17, 18]. Epigenetic clocks produce estimates for biological ageing based on DNA methylation (DNAm) alterations within specific CpG (a cytosine nucleotide followed by a guanine nucleotide) sites and are one of the primary hallmarks of biological ageing [18, 19]. Epigenetic clocks can sum up genetic influences and lifetime burden of lifestyles that predict time-to-death. Although previous studies have suggested that LTPA is associated with decelerated biological ageing [20, 21], more research is required because reliable biological age indicators have only recently become available [22–24].

The main purpose of this study was to identify classes of long-term LTPA patterns and to examine whether these classes differ in terms of biological ageing. Furthermore, we aimed to explore class-specific differences in all-cause mortality, considering biological ageing to be a potential mediator of the favourable associations between long-term LTPA and all-cause mortality. To evaluate the influence of reverse causality, we studied whether the association between long-term LTPA and mortality is affected if the participants with prevalent cardiovascular diseases (CVDs) are excluded, and we investigated the association separately for short-term and long-term mortality. Finally, using a twin study design, we examined whether the associations between long-term LTPA and mortality are independent of shared genetic and environmental factors.

## Materials and methods

### Study design and participants

Participants were twins from the older Finnish Twin Cohort (FTC) [25]. The FTC consists of same-sex twins born in Finland before 1958 and with both co-twins alive in 1967. Questionnaires were mailed in 1975 and 1981 to all twins born before 1958 and living in Finland, with a follow-up questionnaire conducted in 1990 with twins born in 1930–1957. The response rates were high (77–89%). Participants aged 18–50 years at baseline in 1975, who had at least one measurement of LTPA and were alive in 1990, were included in the present study (*n* = 22,750). For the subsample (*n* = 1,153), a blood sample was taken during the 1993–2020 period, and blood-based DNAm was used to assess biological ageing at age range from 37 to 81 years.

### Measurements

#### Leisure-Time Physical Activity (LTPA)

LTPA was measured in metabolic equivalent (MET) hours per day (h/day) using a structured validated questionnaire in 1975, 1981 and 1990 [10, 15, 26, 27] (see Supplementary Tables S1–S2 for details). In 1975 and 1981 questions concerned monthly frequency of LTPA, mean duration and intensity of the sessions (Table S1). The MET index was calculated by multiplying the frequency, intensity, and duration of leisure activities as well as commuting activities, and then summing up the resulting values [15, 26]. In 1990 the questionnaire slightly differed and participants reported their time spent in LTPA (including commuting activity) at different intensity levels (Table S2). The MET index was calculated by multiplying the time spent in LTPA by the estimated MET value of each intensity level and then summing up the resulting values [27].

#### Outcome variables

*Biological ageing* was assessed using blood-based epigenetic ageing measures, namely, principal component (PC)-based DNAm GrimAge [23, 24] and DunedinPACE [22]. DNAm GrimAge is a mortality predictor by design, and it is composite of age, sex, DNAm-based surrogates for seven plasma proteins and smoking pack-years [24]. Recently, PC-based epigenetic clocks have been developed to bolster the reliability and validity of the clocks [23]. The DunedinPACE estimator was developed to predict pace of ageing, describing longitudinal changes in 19 age-related biomarkers [22]. It provides an estimate of the pace of ageing in years per calendar year.

Genomic DNA was extracted from peripheral blood samples using commercial kits. High molecular weight DNA samples (1 µg) were bisulfite converted using EZ-96 DNA /methylation-Gold Kit (Zymo Research, Irvine, CA, USA) according to the manufacturer’s protocol. The twins and co-twins were randomly distributed across plates, with both twins from a pair on the same plate. DNAm profiles were obtained using Illumina’s Infinium HumanMethylation450 BeadChip or the Infinium MethylationEPIC BeadChip (Illumina, San Diego, CA, USA). The Illumina BeadChips measure single-CpG resolution DNAm levels across the human genome. With these assays, it is possible to interrogate over 450,000 (450k) or 850,000 (EPIC) methylation sites quantitatively across the genome at single-nucleotide resolution. Of the samples included in the present study, 419 were assayed using 450k and 734 samples using EPIC arrays. Methylation data from different platforms was separately preprocessed using R package *meffil* [28], and the pipeline for preprocessing was recently described in detail [29]. Beta values representing DNAm levels were used as input in the calculations of the estimates of epigenetic ageing.

We produced PC-based GrimAge estimates using an R package [23] (https://github.com/MorganLevineLab/PC-Clocks). Age acceleration (AA_PC−Grim_) in years was determined as a residual by regressing the estimated epigenetic age on chronological age. In addition, we obtained PC-based components of GrimAge (adjusted for age), including DNAm smoking pack-years, DNAm adrenomedullin (ADM), DNAm beta-2-microglobulin (B2M), DNAm cystatin C, DNAm growth differentiation factor 15 (GDF15), DNAm leptin, DNAm plasminogen activator inhibitor 1 (PAI-1), and DNAm tissue inhibitor metalloproteinases 1 (TIMP-1). The estimates for pace of ageing were calculated using an R package [22] (https://github.com/danbelsky/DunedinPACE). The measures were screened for outliers (> 5 standard deviations away from mean), and there were no outliers.

##### Mortality

Dates of death were retrieved from the Population Register Centre of Finland and Statistics Finland. The mortality follow-up started with the response date to the 1990 questionnaire and continued until the date of emigration, death or 31 December 2020, whichever came first.

#### Confounding variables

Time-varying confounders cause bias for the studies on long-term LTPA and mortality [2]. In our study, on one hand, LTPA was measured over 15-year period and the other lifestyle-related factors may have considerably changed after baseline. The participants were 18 to 50 years old at the baseline, and the education of the youngest participants was still in progress at that time. Moreover, smoking and alcohol use during early adulthood may not reflect their health habits in later life. On the other hand, exposure may have affected the other lifestyle-related factors measured over a long period and thus, these factors may partly mediate rather than confound the association between exposure and outcome [2]. Moreover, our sample included older participants (born in 1925–29) who had information on mortality (*n* = 1,667) and DNAm (*n* = 144) but were not invited to answer questionnaire in 1990. For these reasons, we utilized confounders assessed in 1981 when participants were 24 to 56 years old. Prevalent CVDs, socioeconomic status, and other lifestyle factors (including smoking and alcohol use), as well as body size, were considered as potential confounders. These factors could have affected long-term LTPA and mortality.

##### Health status

An indicator of physician-diagnosed CVD (angina pectoris and myocardial infarction) based on self-reports from 1975 to 1981 was used.

*Education* (in years) was used as proxy for socioeconomic status and was based on the self-reported latest education in 1981 and converted into years of education as follows: less than primary school (4), primary school (6), junior high school (9), high school graduate (12), university degree (17), and ≥ 1 year of education such as vocational training in addition to primary school (8), junior high school (11) or high school (14) [30].

*Body mass index (BMI)* (kg/m^2^) was calculated based on self-reported height and weight in 1981. BMI based on self-reports has been shown to agree well with BMI based on measured values [31].

*Smoking status* was self-reported based on an extensive smoking history [32] and classified as never, occasional, former and current light (1–9 cigarettes per day [CPD]), medium (10–19 CPD) and heavy (≥ 20 CPD) smokers.

*Alcohol use* was based on average alcohol consumption (g/day) in 1981 of beer, wine and spirits [33] and classified as never, former, occasional (> 0.1 and < 1.3 g), low (≥ 1.3 and < 25 g), medium (≥ 25 and < 45 g), high (≥ 45 and < 65 g) and very high (≥ 65 g) [34].

### Statistical analysis

The main analyses were conducted using Mplus 8.2 [35]. The parameters of the models were estimated using the full information maximum method (FIML) with robust standard errors, which uses all available data during estimation.

We conducted latent profile analysis (LPA) to identify long-term patterns of LTPA based on the means and variances of MET indices in 1975, 1981 and 1990. An LPA model with 1–6 classes was fitted. Several indices were used to evaluate the goodness of fit: Akaike’s information criterion, Bayesian information criterion and sample size-adjusted Bayesian information criterion. The lower values of the information criteria indicated a better fit for the model. Furthermore, we used the Vuong–Lo–Mendell–Rubin likelihood ratio (VLMR) test and the Lo–Mendell–Rubin (LMR) test to determine the optimal number of classes. The estimated model was compared with the model with one class less, and the low *p* value suggested that the model with one class less should be rejected. At each step, the classification quality was assessed using the average posterior probabilities for most likely latent class membership (AvePP). AvePP values close to 1 indicate a clear classification. In addition to the model fit, the final model for further analyses was chosen based on the parsimony and interpretability of the classes.

The mean differences between the classes in later biological ageing were studied using the Bolck-Croon-Hagenaars (BCH) approach, which controls for measurement error in classification [36]. The standard errors of the models were corrected for nested sampling within families. The analyses were adjusted for sex, age and timing of the blood drawn (Model 1), and additionally for education, smoking and alcohol use (Model 2) and for BMI (Model 3). The continuous covariates were standardized before entering the model. In addition, differences in the DNAm-based plasma proteins and smoking pack-years between the classes were explored.

**Fig. 1:**
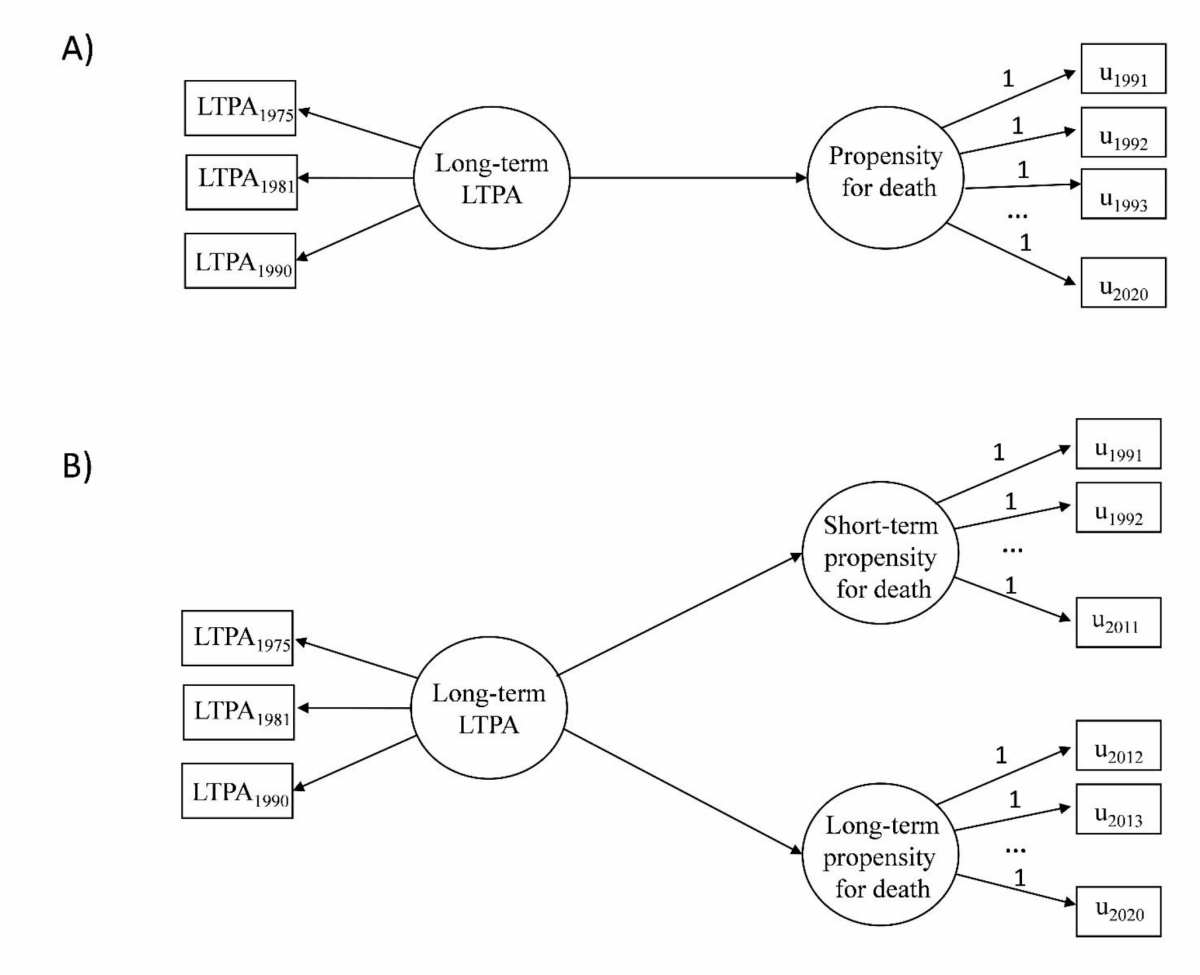
Path diagram of the discrete-time survival models for (**A**) total mortality and (**B**) short- and long-term mortality. Follow-up time was treated as time scale in the analysis. Circles denote latent variables and rectangles observed variables LTPA, leisure-time physical activity; u, discrete-time survival indicators

We investigated differences in total all-cause mortality as well as in short- and long-term all-cause mortality between the classes using a discrete-time survival model, which enables flexible modelling within the structural equation framework [37, 38]. We used year as the unit of discrete-time survival indicators from 1991 to 2020 and constructed a latent variable describing propensity for death [37, 38] (see Fig. 1A). The latent variable was regressed on the latent class membership of long-term LTPA and the potential confounding variables. Moreover, we divided the follow-up time into two parts, formed latent variables representing propensity for short- and long-term death, and the associations of long-term LTPA on short- and long-term mortality were studied (Fig. 1B). This approach relaxes strict proportionality assumption often necessary in the survival modeling, by allowing the associations of exposure with short- and long-time mortality to vary. The model was adjusted similarly to the models of biological aging. However, we also excluded the participants who reported prevalent CVD (angina pectoris or myocardial infarction) and rerun the analyses. The best practices in relating LPA to time-to-event distal outcomes are still under research [39, 40]. Because BCH approach was not available for the survival models, we used posterior classification probabilities as sampling weights to control for measurement error in the classification (three-step approach with proportional assignment) [39]. The standard errors of the model parameters were corrected for nested sampling within families.

To account for familial factors, the associations between long-term LTPA and total all-cause mortality were studied at the within-twin-pair level using multilevel modelling. For monozygotic (MZ) twin pairs, the association was controlled for shared environmental factors and genetics, while for dizygotic (DZ) pairs, it was controlled for shared environmental factors and partially for genetics.

We investigated the association between biological ageing and total all-cause mortality using a discrete-time survival model, as well. For these analyses, mortality follow-up began from the time of blood draw. To account for late entry, the discrete-time survival indicators were recoded as missing values for time points before an individual entered the study.

## Results

The descriptive statistics of the study variables are presented in Table 1 and are stratified by sex in Supplementary Table S3.

**Table 1:** Descriptive statistics of the study variables for all twins and the subsample of twins with information on biological ageing

	All twins (*n* = 22,750)		Subsample (*n* = 1,153)	
	*n*	Mean (SD) or %	*n*	Mean (SD) or %
Sex				
Female	11,308	49.7	933	80.9
Male	11,442	50.3	220	19.1
Age at baseline	22,750	30.0 (8.9)	1,153	33.2 (9.7)
Zygosity				
Unsure	1,902	8.4	-	
Monozygotic	6,462	28.4	610	52.9
Same-sex dizygotic	14,386	63.2	543	47.1
Health status				
Cardiovascular diseases^a^	20,588		1,111	
No	19,726	95.8	1,073	96.6
Yes	862	4.2	38	3.4
Latest education in 1981 (in years)	22,205	8.5 (3.2)	1,131	9.2 (3.5)
**Leisure-time physical activity**				
Metabolic equivalent (MET) index (h/day)				
in 1975	20,984	2.4 (3.1)	1,116	2.2 (2.5)
in 1981	20,165	2.6 (3.1)	1,098	2.4 (2.4)
in 1990	12,312	3.3 (3.4)	841	3.1 (2.8)
**Lifestyle-related factors in 1981**				
Body mass index (kg/m^2^)	20,074	23.5 (3.4)	1,097	23.4 (3.4)
Smoking	19,850		1,153	
Never	8,932	45.0	703	64.8
Occasional	624	3.1	33	3.0
Former	4,149	20.9	182	16.8
Light	1,476	7.4	53	4.9
Medium	2,714	13.7	77	7.1
Heavy	1,955	9.8	37	3.4
Alcohol use^b^	19,818		1,091	
Never	1,768	8.9	167	15.3
Former	1,092	5.5	69	6.3
Occasional	1,045	5.3	77	7.1
Low	14,337	72.3	746	68.4
Medium	961	4.8	20	1.8
High	398	2.0	10	0.9
Very high	217	1.1	2	0.2
**Outcomes**				
Deaths (1991–2020)	6,949	30.6	267	23.2
Biological ageing				
Age at blood drawn	-		1,153	63.5 (9.1)
PC-based DNAm GrimAge (in years)			1,153	70.9 (7.3)
DunedinPACE (year/calendar year)	-		1,153	0.98 (0.12)

SD, standard deviation; DNAm, DNA methylation; PC, principal component

^a^Self-reported physician-diagnosed angina pectoris or myocardial infarction in 1975 or 1981

^b^High and very high classes were combined for further analysis

### Patterns of long-term LTPA

**Fig. 2:**
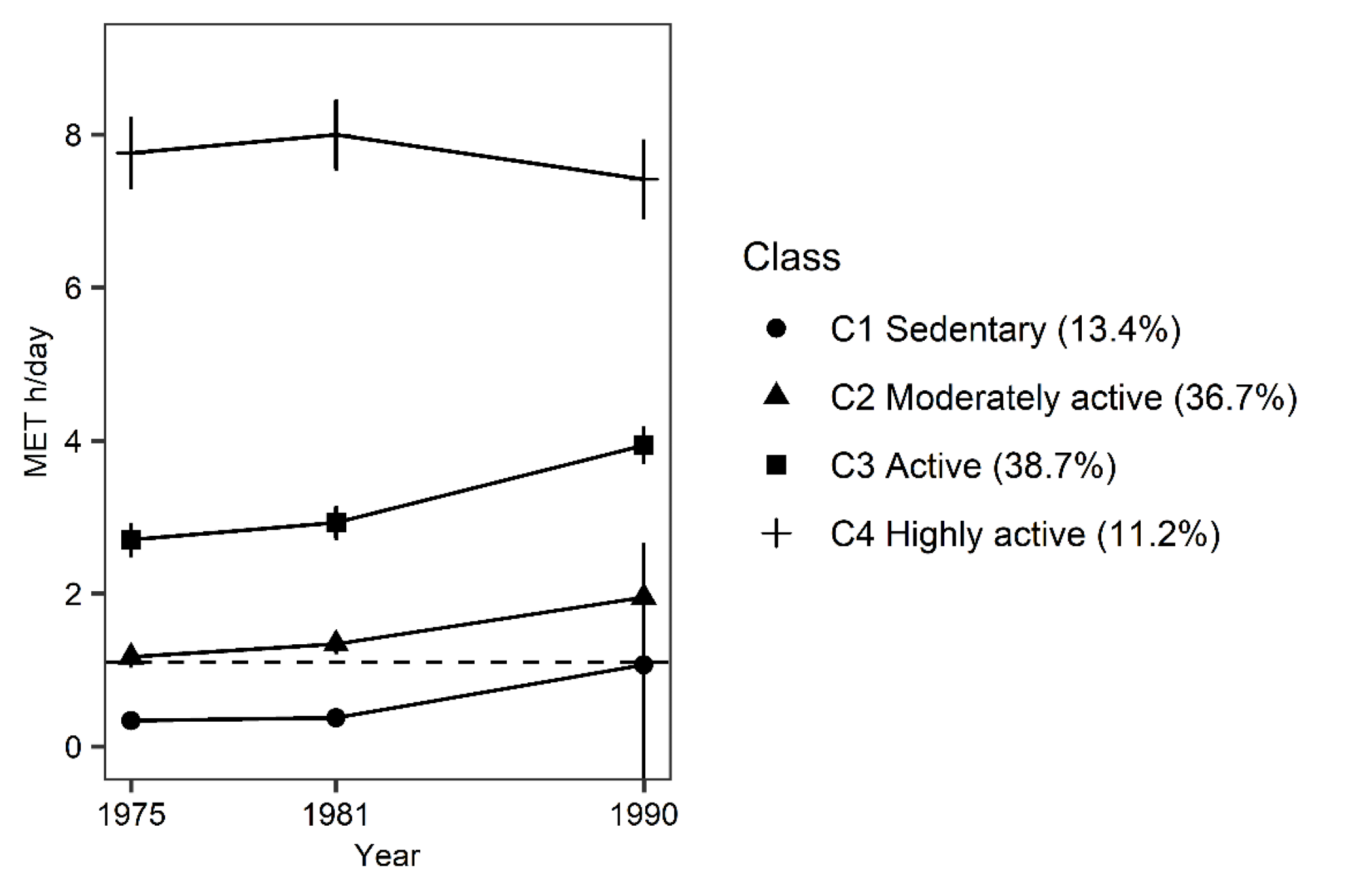
Patterns of long-term leisure-time physical activity (*n* = 22,750)

The model-fit based on the information criteria improved at each step (Supplementary Table S4). However, at the sixth step, only a small class (< 5%) was extracted, and therefore, a solution with 4–5 classes was considered optimal. At the fifth step, a class with increasing LTPA pattern from sedentary to moderate level was identified. To achieve sufficient power for the further analysis, we used a four-class solution in the main analyses (Fig. 2). The level of LTPA appeared to increase slightly between years 1981 and 1990, except in the highly active class. This is probably due to the small changes in the questionnaire (see Supplementary Tables S1–S2) rather than reflecting actual increase in LTPA. For sensitivity analysis, we conducted the main analyses using a five-class solution (Supplementary Fig. S3–S5).

Means of metabolic equivalent (MET) hours (h)/day and 95% confidence intervals are presented. The dashed line denotes World Health Organization guidelines for the recommended minimum amount of physical activity for adults (150 min of moderate intensity physical activity per week ~ 1.1 MET h/day) [5].

### Differences in biological ageing between long-term LTPA classes

A high correlation was observed between chronological age and biological age assessed with PC-DNAm GrimAge estimator (*r* = 0.85). In contrast, the correlation between chronological age and pace of ageing assessed with DunedinPACE was low (*r* = 0.19), as expected. The correlation between DunedinPACE and AA_PC-Grim_ estimator was high (*r* = 0.61).

**Fig. 3:**
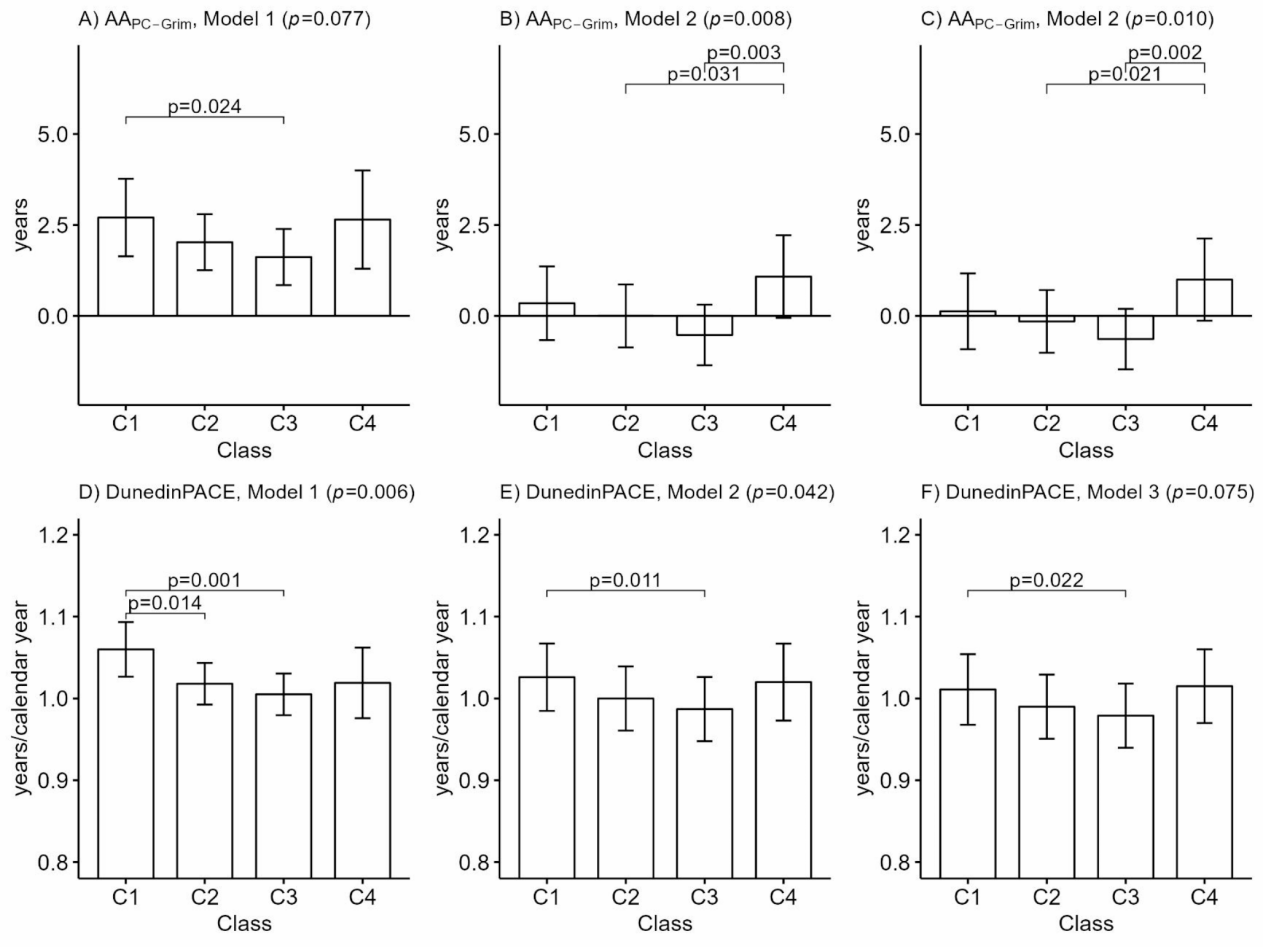
Mean differences between the long-term leisure-time physical activity classes in terms of biological ageing measured using (**A**)–(**C**) PC-based GrimAge and (**D**)–(**F**) DunedinPACE (*n* = 1,153)

There were differences between the classes in terms of biological ageing, measured using AA_PC-Grim_ and DunedinPACE (Fig. 3). The association between long-term LTPA and biological ageing followed a U-shaped pattern: participants in the sedentary and highly active classes were biologically older than those who were moderately active and active. After adjusting for other lifestyle-related factors, most differences were attenuated, but based on AA_PC-Grim_, the highly active class remained, on average, 1.2 years (95% confidence interval: 0.2–2.2) biologically older than the moderately active class and 1.6 years (0.6–2.7) biologically older than the active class. As no significant beneficial association between long-term LTPA and slower biological ageing was observed, biological ageing was unlikely to act as a mediator for the association between long-term LTPA and lower mortality, and no further path modelling was conducted.

Of the PC-GrimAge components, the U-shaped association was most pronounced in DNAm-based cystatin C and B2M (Supplementary Fig. S1). There were also differences in DNAm-based smoking pack-years. In the highly active class, the level was higher than in the other classes, which may indicate under-reporting in the highly active class because the models were adjusted for self-reported smoking. There were no differences in DNAm ADM, GDF15, leptin, PAI-1 or TIMP-1.

Means and 95% confidence intervals are presented. Model 1 was adjusted for sex, age and timing of the blood drawn. Model 2 was additionally adjusted for education, smoking and alcohol use, and Model 3 additionally for body mass index. C1: Sedentary (8.8%); C2: Moderately active (38.4%); C3: Active (45.5%); C4: Highly active (7.3%); AA, Age acceleration; *p*-values from the Wald test.

### Differences in mortality between the latent long-term LTPA classes

**Fig. 4:**
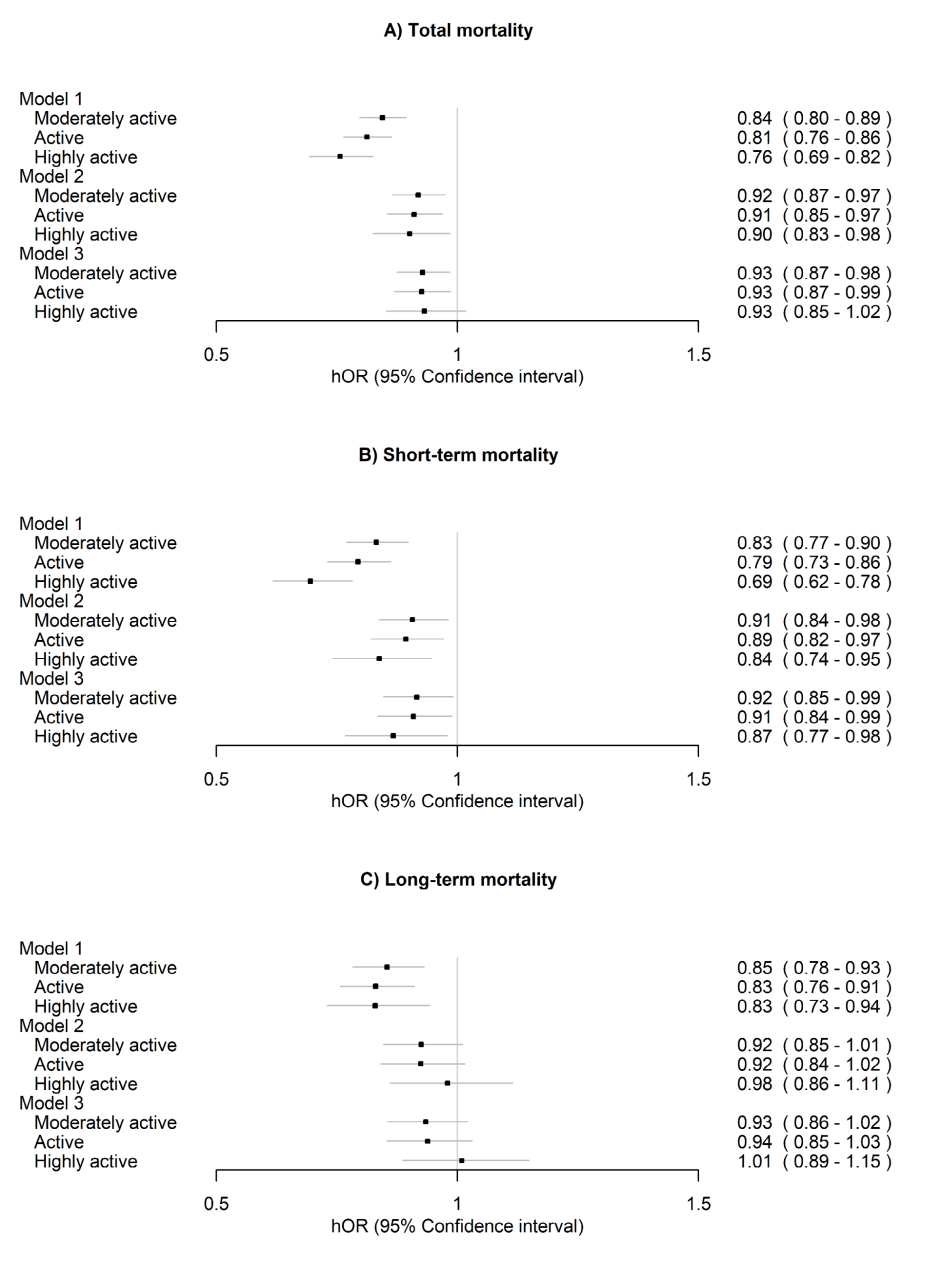
Associations of long-term leisure-time physical activity with (**A**) total mortality, (**B**) short-term mortality (1990–2011) and (**C**) long-term mortality (2012–2020) (*n* = 22,750)

Over a third (38.8%) of the participants from the sedentary class died during the mortality follow-up period, compared to 30.8%, 29.0% and 25.4% from the more active classes, respectively. Active classes had 16–24% lower all-cause mortality risk compared to the sedentary class, but after accounting for other lifestyle-related factors (Model 2) and additionally for BMI (Model 3), the reduction in mortality risk was a maximum of 9% and 7%, respectively (Fig. 4A). Approximately half of the deaths occurred in 2011 or earlier. Therefore, 2011 was considered the cut-off point for short- and long-term mortality (Fig. 4B–C). For sensitivity analysis, we performed the modelling using 2006 as the cut-off, when one-third of the deaths had occurred (Supplementary Fig. S6). Overall, the favourable associations of long-term LTPA were more consistent with short-term than long-term mortality. In particular, high activity was associated only with lower risk of short-term mortality. The interpretation of the results was similar after excluding participants with prevalent CVD, but the associations with long-term LTPA and mortality were further attenuated (Supplementary Fig. S2).

The sedentary class was treated as the reference. Model 1 was adjusted for sex (female) and age. Model 2 was additionally adjusted for education, smoking and alcohol use and Model 3 additionally for body mass index. hOR, hazard odds ratio.

### Within-twin-pair differences in mortality between long-term LTPA classes

**Fig. 5:**
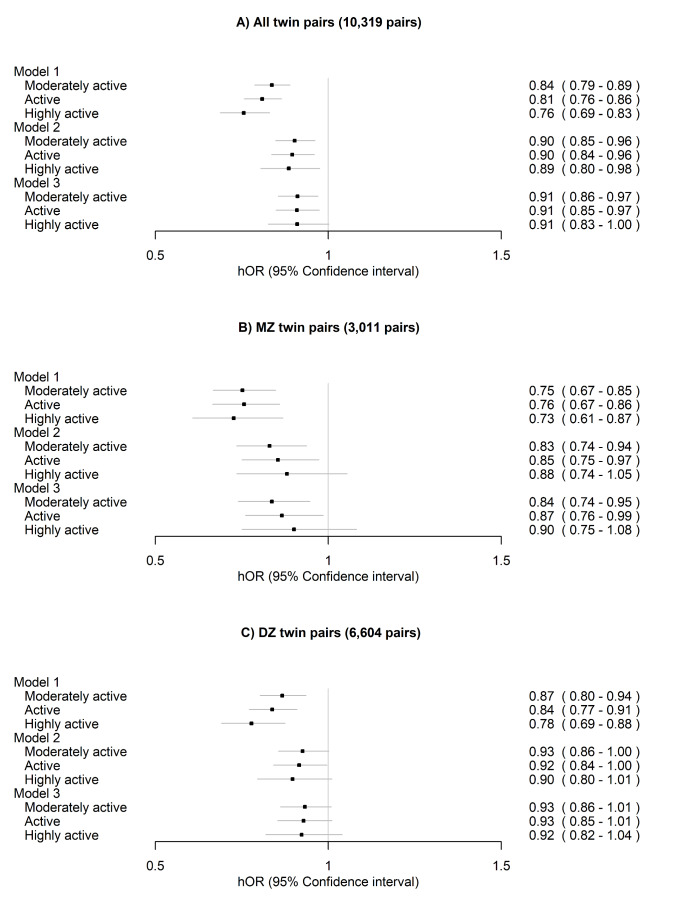
Within-twin-pair differences in all-cause mortality between the long-term leisure-time physical activity classes for (**A**) all twin pairs, (**B**) monozygotic (MZ) pairs and (**C**) dizygotic (DZ) pairs. The sedentary class was treated as the reference. Only twin pairs with information on LTPA and alive in 1990 were included in the analysis. Model 1 was adjusted for sex (female) and age at the between-twin-pair level. Model 2 was additionally adjusted for education, smoking and alcohol use at the within-twin pair level and Model 3 for body mass index at within-twin pair level. hOR, hazard odds ratio

Before any adjustments at within-twin-pair level, the analyses showed that the moderately active and active classes exhibited lower risks of all-cause mortality compared to the sedentary class within all pairs, DZ pairs and MZ pairs (Fig. 5, Model 1). After additionally adjusting the model for other lifestyle-related factors, the differences were considerably attenuated, but the moderately active and active classes exhibited lower risks of all-cause mortality compared to the sedentary class within all pairs, DZ pairs and MZ pairs and highly active class within all pairs (Fig. 5, Model 2). Including BMI in the models only slightly attenuated the differences (Fig. 5, Model 3).

**Fig. 6:**
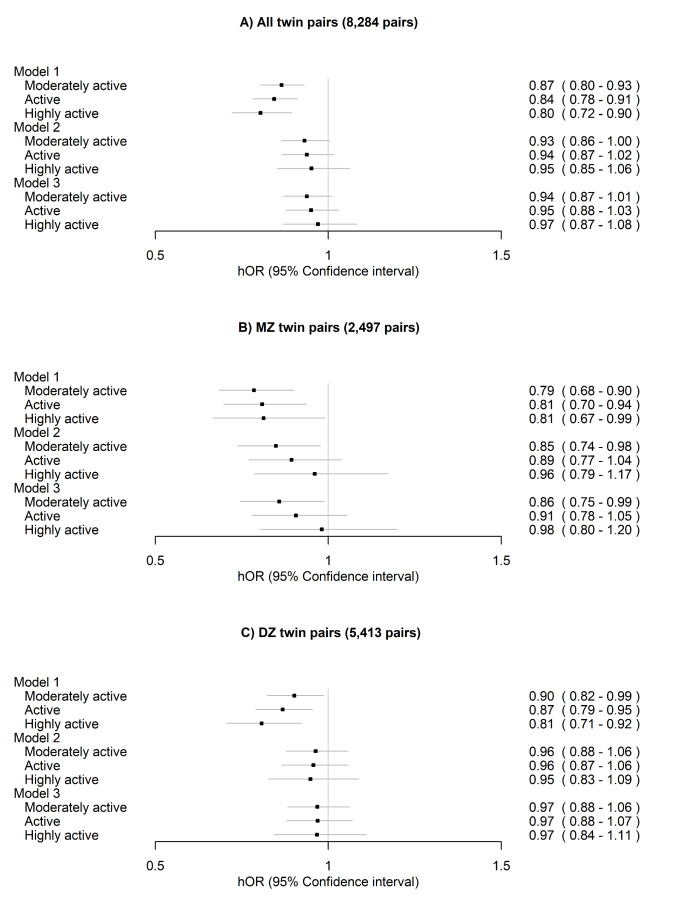
Within-twin-pair differences in all-cause mortality between the long-term leisure-time physical activity classes for (**A**) all twin pairs, (**B**) monozygotic (MZ) pairs and (**C**) dizygotic (DZ) pairs after excluding twin pairs who reported prevalent cardiovascular disease (angina pectoris or myocardial infarction in 1975 or 1981). The sedentary class was treated as the reference. Only twin pairs with information on LTPA and alive in 1990 were included in the analysis. Model 1 was adjusted for sex (female) and age at the between-twin-pair level. Model 2 was additionally adjusted for education, smoking and alcohol use at the within-twin-pair level and Model 3 for body mass index at the within-twin-pair level

After excluding the twin pairs who reported prevalent CVD, the differences in all-cause mortality were further attenuated and were no longer significant after adjusting for other lifestyle-related factors and additionally for BMI for all twin pairs, MZ pairs and DZ pairs (Fig. 6, Model 2 and Model 3). However, the difference between the moderately active and the sedentary class remained significant within MZ pairs.

### Association between biological ageing and all-cause mortality

After adjusting for sex and age, one standard deviation (SD) increase in AA_PC-Grim_ was associated with 49% increased mortality (hOR = 1.49, 95% confidence interval CI 1.29–1.74) and one SD increase in DunedinPACE was associated with 31% increased mortality (hOR = 1.31, 95% CI 1.13–1.52). After additionally adjusting for LTPA, the results remained the same (hOR = 1.50, 95% CI 1.29–1.74 and hOR = 1.31, 95% CI 1.35–1.52, respectively).

### Sensitivity analysis using a five-class solution

After including fifth class in the LCA model, a class of increasingly active (from sedentary to moderate) participants was extracted (Supplementary Fig. S3). According to the overall test there were differences between the classes in biological ageing measured with AA_PC−Grim_ but not in DunedinPACE (Supplementary Fig. S4). Biological ageing measured with AA_PC−Grim_ appeared to be accelerated in highly active class. There were differences in mortality between the classes, but the differences were smaller than observed in the main analysis (Supplementary Fig. S5). Increasingly active class did not differ from sedentary class in terms of mortality risk. After accounting for other health-related factors, there were differences only in short-term mortality.

## Discussion

We conducted a 30-year mortality follow-up of a longitudinal study of LTPA with a large cohort of adult twins and identified classes according to long-term LTPA patterns. Our analyses were based on standard cohort approaches to individuals (adjusting for the sampling of twin pairs) and on within-pair modelling to account for familial factors. The results showed that the beneficial associations of long-term LTPA with slow biological ageing and reduced mortality were largely accounted for by other health-related factors. The most remarkable reduction of 7% in all-cause mortality was observed already when the recommended minimum amount of LTPA was, on average, achieved, with no additional benefits provided by higher levels of LTPA. This result is in line with the World Health Organization guidelines [5] and studies demonstrating that dose-response association between physical activity (PA) and all-cause mortality is curvilinear rather than linear [3, 4, 41]. The association between long-term LTPA and all-cause mortality was susceptible to reverse-causality bias because a consistent association was observed only in the short term. Moreover, the within-twin-pair differences attenuated when twin pairs reporting prevalent CVD were excluded. An alternative explanation is a change in PA exposure as a function of time; in other words, some people may have changed their PA levels later in adulthood.

### The association between long-term LTPA and mortality was susceptible to bias from multiple sources

Previous studies of long-term LTPA have reported larger differences in mortality between consistently active and inactive participants (16–36%) [42–44] than what we observed in our study. This discrepancy can be explained by differences in the length of the mortality follow-up time; longer follow-up time has been shown to be associated with weaker associations between PA and mortality [11]. In our study, participants were followed over a 30-year period. Therefore, we were able to divide the follow-up time into two parts to reflect short- and long-term survival. Our analysis revealed that the associations of long-term LTPA were more consistent with short-term than long-term mortality. Being highly active was associated with reduced mortality only in the short term and thus may not have long-term mortality benefits unless activity is maintained continuously.

Another reason for the higher effect sizes observed in previous studies may be residual confounding due to insufficient adjustments. In our study, when the models were minimally adjusted, the reduction in mortality (16–24%) was closer to the level observed in previous studies. After adjustment for smoking in terms of both status and quantity, and other lifestyle-related factors (education and alcohol use, and additionally for BMI), the association was considerably attenuated. Smoking is the most harmful lifestyle habit in terms of mortality [45, 46]. Often, only smoking status is adjusted for in analyses, but this may not be sufficient because current smokers who are physically active tend to smoke less than those who are sedentary (Supplementary Table S5).

Previous twin studies have provided somewhat inconsistent results regarding the association between LTPA and mortality after accounting for familial factors. Studies using data from Finnish twins have suggested that the association between LTPA and all-cause mortality is due to genetic selection, as there was no difference in mortality between MZ co-twins discordant in terms of LTPA [10, 46], whereas a study with a similar twin study setting found a difference within Swedish twin pairs [47]. Several reasons for this discrepancy have been proposed, such as differences in LTPA measurement methods and in controlling for prevalent diseases [48]. Our within-twin-pair comparisons were considerably affected by how the prevalent diseases were controlled for. Excluding twin pairs with one or both co-twins reporting diseases (as done in prior Finnish studies) strongly attenuated the differences, particularly when other lifestyle-related factors were controlled for. This finding may support studies arguing that excluding participants with diseases at baseline is necessary to mitigate reverse causation [12] and may also reflect an accumulation of unhealthy lifestyle habits and prevalent CVD in the sedentary class (Supplementary Table S5).

### Long-term LTPA and biological ageing

Previous studies, mostly based on cross-sectional data, have indicated that the observed associations, or lack of associations, between LTPA and biological ageing depend on the type of epigenetic clock used [20, 49–53]. This is probably because the first-generation clocks were developed to predict chronological age [54, 55], while newer clocks, such as DNAm GrimAge, are better predictors of health and lifespan [24, 56]. LTPA is most consistently associated with biological ageing, assessed using the DNAm GrimAge estimator. However, the association may vary by ethnicity, as no association was observed in African or African American population [53, 57]. To the best of our knowledge, this is the first study to report on the association between LTPA and DunedinPACE. We found that biological ageing was, on average, slower in the active class compared to the sedentary class when these two markers of biological ageing were used. However, after adjusting the model for other lifestyle-related factors, the differences were largely attenuated, which likely reflects an accumulation of factors related to an unhealthy lifestyle in the sedentary class [58]. Moreover, the weak associations observed in the present study may be due to the prospective study design, as the beneficial influences of LTPA may diminish after a time lag of several years.

Contrary to the existing literature [20, 50, 52], we observed that the highly active class was, on average, biologically older than the moderately active and active classes when the DNAm (PC) GrimAge was used. However, a recent study indicated a curvilinear association between accelerometer-based PA and biological ageing [51]. Some studies have reported U-shaped associations between LTPA and mortality [59], and it has been suggested that sudden cardiac deaths after/during exercise may explain the increased mortality at high levels of LTPA [8, 59]. However, a recent meta-analysis did not find evidence of increased levels of mortality at high PA levels [1].

To better understand the reasons for the observed patterns, we explored the DNAm-based plasma proteins included in the DNAm GrimAge estimator. Interestingly, the levels of DNAm-based cystatin C and beta-2-microglobulin (B2M) were higher in both the sedentary and highly active classes. These proteins are markers of kidney function, and their higher concentrations are linked to mortality due to cardiovascular diseases [60] and sudden cardiac death [61]. Further studies are required to determine whether these DNAm-based proteins play a role in sudden cardiac death in athletes.

### Strengths and limitations

Our study has major strengths. We used prospective population-based large cohort data with longitudinal measurements of LTPA over 15 years with validated questionnaires and 30 years of mortality follow-up. The LTPA patterns were studied using a data-driven method without using preselected cut-offs. Biological ageing was assessed using novel epigenetic clocks, which have been shown to perform better than their predecessors [22, 23]. The twin study design enabled us to control the analyses for genetics and the shared environment.

However, our study also had limitations. In the subsample of twins having information on biological ageing, women and non-smokers were overrepresented, alcohol use was lower, and there were fewer deaths among men (see Supplementary Table S3), which may limit the generalizability of our findings to the general population. Otherwise, the subsample represented the larger cohort relatively well. LTPA was self-reported, and a considerable proportion of the participants did not have LTPA at all three measurement points. Self-reports may be susceptible to recall and social-desirability biases. However, the questionnaire-based MET index has been shown to be a reliable tool for measuring LTPA [15], and self-reports may better reflect long-term LTPA than device-based measures. Although twin design efficiently controls for shared confounders including genetic factors and socioeconomic background during childhood, there may be influences of non-shared unmeasured confounders (e.g. dietary and occupational exposures) which were not controlled in the analyses.

## Conclusions

Our findings suggest that the association between LTPA and all-cause mortality is susceptible to bias from multiple sources including residual confounding, reverse causality and genetic confounding. The association between LTPA and lower all-cause mortality may be largely due to a healthy phenotype and an overall healthy lifestyle co-occuring with high levels of LTPA and a lower mortality risk. When considering biological ageing and all-cause mortality, following an overall healthy lifestyle may be more beneficial than maintaining high levels of LTPA. Further research on DNAm-based surrogates may provide insights into the mechanisms behind the beneficial and detrimental health influences of LTPA.

## Electronic supplementary material

Below is the link to the electronic supplementary material.

Supplementary Material 1

## Declarations

### Data sharing

A subsample of the FTC with DNA methylation age estimates, phenotypes and information on the classes of long-term LTPA will be located in the Biobank of the National Institute for Health and Welfare. All these data will be publicly available for use by qualified researchers following a standardised application procedure (for details on the application process, see the following website: https://thl.fi/en/web/thl-biobank/for-researchers). Because of the consent given by the study participants and the high degree of identifiability of the twin siblings in Finland, the full cohort data cannot be made publicly available. However, the full cohort data are available through the Institute for Molecular Medicine Finland (FIMM) Data Access Committee (DAC) for authorised researchers with an IRB/ethics approval and an institutionally approved study plan. For more details, please contact the FIMM DAC (fimm-dac@ helsinki.fi).
