# Supplementary material for: The associations of long-term physical activity in adulthood with later biological ageing and all-cause mortality – a prospective twin study
Source: Eur J Epidemiol. 2025 Jan 17;40(1):107–22. doi: 10.1007/s10654-024-01200-x (PMC11799114; doi:10.1007/s10654-024-01200-x)
Supplement: Supplementary file 1 — Supplementary Material 1 [file 10654_2024_1200_MOESM1_ESM.docx]

The associations of long-term physical activity in adulthood with later biological ageing and all-cause mortality – a prospective twin study

**Supplementary material –** Kankaanpää et al.

**Supplementary tables**

[**Table S1**. Physical activity questionnaire in 1975 and 1981 and scoring for MET index. 2](#_Toc149144368)

[**Table S2.** Physical activity questionnaire in 1990 and scoring for metabolic equivalent (MET) index. 3](#_Toc149144369)

[**Table S3.** Descriptive statistics of the study variables stratified by sex for all twins and the subsample of twins with information on biological ageing. 4](#_Toc149144370)

[**Table S4.** Model fit of the latent profile models with different number of classes (n = 22,750). 6](#_Toc149144371)

[**Table S5.** The characteristics in the latent classes with different long-term leisure-time physical activity patterns in the population weighted by classification probabilities (n = 22,750). 7](#_Toc149144372)

**Supplementary figures**

[**Fig. S1** Mean differences in PC-based DNA methylation (DNAm)-based plasma proteins and smoking pack-years between the classes of long-term leisure-time physical activity (n = 1,153): A) DNAm adrenomedullin (ADM), B) DNAm beta-2 microglobulin (B2M), C) DNAm growth differentiation factor (GDF15), D) DNAm cystatin C, E) DNAm leptin, F) DNAm plasminogen activation inhibitor 1 (PAI-1), DNAm tissue inhibitor metalloproteinase 1 (TIMP-1), and H) DNAm smoking pack-years (packyrs).. 8](#_Toc149144291)

[**Fig*.*** **S2** Associations of long-term leisure-time physical activity with A) total mortality, B) short-term mortality (1990–2011), and C) long-term mortality (2012–2020). Twins who did not report selected diseases were included in the analysis (n = 19,726). 9](#_Toc149144292)

[**Fig. S3** Sensitivity analysis: Latent profile solution with five classes (n = 22,750).. 10](#_Toc149144293)

[**Fig. S4** Sensitivity analysis: Latent profile solution with five classes; mean differences between the classes of long-term leisure-time physical activity in biological ageing measured with A–C) PC-based GrimAge and D–F) DunedinPACE (n=1,153). 11](#_Toc149144294)

[**Fig. S5** Sensitivity anaysis: Latent profile solution with five classes; the associations of long-term leisure-time physical activity with A) total mortality, B) short-term mortality (1990–2011), and C) long-term mortality (2012–2020) (n=22,750). 12](#_Toc149144295)

[**Fig. S6** Sensitivity analysis: The associations of long-term leisure-time physical activity with A) total mortality, B) short-term mortality (1990–2006), and C) long-term mortality (2007–2020) (n=22,750). 13](#_Toc149144296)

Table S*1*. Physical activity questionnaire in 1975 and 1981 and scoring for MET index.

| **LEISURE ACTIVITY** |  |
| --- | --- |
| **Intensity:** Is your physical activity during leisure-time about as strenuous on average as: | |
|  | Score (MET) |
| a) walking | 4 |
| b) alternately walking and jogging | 6 |
| c) jogging | 10 |
| d) running | 13 |
|  |  |
| **Duration:** How long does the physical activity last at one session on average? | |
|  | Score (min) |
| a) Less than 15 minutes | 7.5 |
| b) 15 min – less than 30 min | 22.5 |
| c) 30 min – less than 1 hour | 45 |
| d) 1 hour – less than 2 hours | 90 |
| e) Over two hours | 120 |
|  |  |
| **Frequency:** Presently how many times per month do you engage in physical activity during your leisure time? | |
|  | Score (times per month) |
| a) less than once a month | 0.5 |
| b) 1-2 times per month | 1.5 |
| c) 3-5 times per month | 4 |
| d) 6-10 times per month | 8 |
| e)11-19 times per month | 15 |
| f) more than 20 times per month | 20 |
|  |  |
| **COMMUTING ACTIVITY** |  |
| **Intensity** |  |
|  | Score (MET) |
|  | 4 |
| **Duration** |  |
| How much of your daily journey to work is spent in walking, cycling, running and/or cross-country skiing? | |
|  | Score (min) |
| a) Less than 15 minutes | 7 |
| b) 15 min – less than a half an hour | 22 |
| c) half an hour to less than an hour | 45 |
| d) hour or more | 75 |
| e) I am presently not at work | 0 |
|  |  |
| **Frequency** |  |
|  | Score (times per month) |
|  | 20 (5×4) |

Table S2. Physical activity questionnaire in 1990 and scoring for metabolic equivalent (MET) index.

| Following question are about your physical activity during leisure time or during your daily journey to work during last 12 months. How many hours in week you engage in physical activity corresponding to each intensity level? | |
| --- | --- |
|  |  |
| **Intensity levels** | Score (MET) |
| Walking | 4 |
| Alternately walking and jogging | 6 |
| Jogging | 10 |
| Running | 13 |
|  |  |
| **Duration** |  |
|  | Score (min per month) |
| a) Not at all | 0 |
| b) Less than 30 min per week | 60 (15×4) |
| c) 30 min – less than 1 hour per week | 180 (45×4) |
| d) 2-3 hours per week | 600 (150×4) |
| e) 4 hours or more per week | 960 (240×4) |

Table S3. Descriptive statistics of the study variables stratified by sex for all twins and the subsample of twins with information on biological ageing.

|  | All twins (n = 22,750) | |  |  | Subsample (n = 1,153) | |  |  |
| --- | --- | --- | --- | --- | --- | --- | --- | --- |
|  | Men (n = 11,308) | | Women (n = 11,442) | | Men (n = 220) | | Women (n = 933) | |
|  | n | Mean (SD) or % | n | Mean (SD) or % | n | Mean (SD) or % | n | Mean (SD) or % |
| Age at baseline | 11,308 | 30.2 (8.9) | 11,442 | 29.8 (9.0) | 220 | 26.2 (5.6) | 933 | 34.8 (9.7) |
| Zygosity |  |  |  |  |  |  |  |  |
| Unsure | 1,030 | 9.1 | 868 | 7.6 | - |  | - |  |
| Monozygotic | 2,993 | 26.5 | 3,469 | 30.3 | 152 | 69.1 | 457 | 49.0 |
| Same-sex dizygotic | 7,285 | 64.4 | 7,105 | 62.1 | 68 | 30.9 | 476 | 51.0 |
| Health status |  |  |  |  |  |  |  |  |
| Cardiovascular diseases ^a^ | 10,100 |  | 10,426 |  | 215 |  | 894 |  |
| No | 9,819 | 97.2 | 10,191 | 97.7 | 215 | 100 | 872 | 97.5 |
| Yes | 281 | 2.8 | 235 | 2.3 | 0 | 0 | 22 | 2.5 |
| **Leisure-time physical activity** |  |  |  |  |  |  |  |  |
| Metabolic equivalent (MET) index |  |  |  |  |  |  |  |  |
| in 1975 | 10,328 | 2.7 (3.6) | 10,656 | 2.1 (2.5) | 216 | 2.8 (3.6) | 900 | 2.0 (2.1) |
| in 1981 | 9,788 | 2.9 (3.7) | 10,377 | 2.3 (2.5) | 210 | 2.6 (2.9) | 888 | 2.3 (2.2) |
| in 1990 | 5,621 | 3.2 (3.6) | 6,691 | 3.3 (3.2) | 173 | 3.4 (3.2) | 668 | 3.0 (2.6) |
| **Health-related factors in 1981** |  |  |  |  |  |  |  |  |
| BMI | 9,768 | 24.4 (3.0) | 10,306 | 22.7 (3.2) | 208 | 22.7 (3.4) | 889 | 23.3 (3.5) |
| Smoking | 11,308 |  | 11,442 |  | 209 |  | 933 |  |
| Never | 3,043 | 31.5 | 5,889 | 57.8 | 93 | 44.5 | 610 | 69.6 |
| Occasional | 384 | 4.0 | 240 | 2.4 | 13 | 6.2 | 20 | 2.3 |
| Former | 2,487 | 25.7 | 1,662 | 16.3 | 52 | 24.9 | 130 | 14.8 |
| Light | 581 | 6.0 | 895 | 8.8 | 10 | 4.8 | 43 | 4.9 |
| Medium | 1,632 | 16.9 | 1,082 | 10.6 | 25 | 12.0 | 52 | 5.9 |
| Heavy | 1,538 | 15.9 | 417 | 4.1 | 16 | 7.7 | 21 | 2.4 |
| Alcohol use ^b^ | 9,672 |  | 10,146 |  | 210 |  |  |  |
| Never | 462 | 4.8 | 1,306 | 12.9 | 10 | 4.8 | 157 | 17.8 |
| Former | 329 | 3.4 | 763 | 7.5 | 9 | 4.3 | 60 | 6.8 |
| Occasional | 182 | 1.9 | 863 | 8.5 | 2 | 1.0 | 75 | 8.5 |
| Low | 7,299 | 75.5 | 7,038 | 69.4 | 169 | 80.5 | 577 | 65.5 |
| Medium | 836 | 8.6 | 125 | 1.2 | 13 | 6.2 | 7 | 0.8 |
| High | 361 | 3.7 | 37 | 0.4 | 6 | 2.9 | 4 | 0.5 |
| Very high | 203 | 2.1 | 14 | 0.1 | 1 | 0.5 | 1 | 0.1 |
| **Outcomes** |  |  |  |  |  |  |  |  |
| Deaths (1991-2020) | 4,291 | 37.9 | 2,664 | 23.3 | 12 | 5.5 | 255 | 27.3 |
| Biological ageing |  |  |  |  |  |  |  |  |
| Age at blood-draw | - |  | - |  | 220 | 65.0 (8.5) | 933 | 63.2 (9.2) |
| PC-based DNAm GrimAge |  |  |  |  | 220 | 74.7 (7.3) | 933 | 70.0 (7.1) |
| DunedinPACE | - |  | - |  | 220 | 1.02 (0.13) | 933 | 0.97 (0.11) |
| SD, standard deviation; DNAm, DNA methylation; PC, Principal component. | | | | |  |  |  |  |
| ^a^ Self-reported physician-diagnosed angina pectoris or myocardial infarction in 1975 or 1981. | | | | | | |  |  |
| ^b^ High and very high classes were combined for further analysis. | | | |  |  |  |  |  |

Table S4. Model fit of the latent profile models with different number of classes (n = 22,750).

| Classes | AIC | BIC | ABIC | VLMR | LMR | Class sizes | AvePP |
| --- | --- | --- | --- | --- | --- | --- | --- |
| 1 | 275443 | 275491 | 275472 | - | - | - | - |
| 2 | 232782 | 232887 | 232845 | <0.001 | <0.001 | 76.8%, 23.2% | 0.95, 0.93 |
| 3 | 221285 | 221446 | 221382 | <0.001 | <0.001 | 54.6%, 29.9%, 15.4% | 0.92, 0.84, 0.95 |
| 4 | 217829 | 218046 | 217960 | 0.227 | 0.230 | 38.7%, 36.7%, 13.4%, 11.1% | 0.86, 0.81, 0.78, 0.93 |
| 5 | 215064 | 215337 | 215229 | <0.001 | <0.001 | 34.9%, 34.2%, 12.7%, 10.0%, 8.3% | 0.79, 0.85, 0.73, 0.93, 0.77 |
| 6 | 212645 | 212974 | 212844 | 0.003 | 0.004 | 38.7%, 33.0%, 11.4%, 8.4%, 6.0%, 2.4% | 0.86, 0.78, 0.92, 0.82, 0.80, 0.94 |
| AIC, Akaike’s information criterion; BIC, Bayesian information criterion; ABIC, sample size-adjusted Bayesian information criterion; VLMR, Vuong-Lo-Mendell-Rubin likelihood ratio test; LMR, Lo-Mendell-Rubin-adjusted likelihood ratio test; AvePP, average posterior probabilities for most likely latent class membership. | | | | | | | |

Table S5. The characteristics in the latent classes with different long-term leisure-time physical activity patterns in the population weighted by classification probabilities (n = 22,750)

|  | C1 Sedentary (13.4%) | | C2 Moderately active (36.7%) | | C3 Active (38.7%) | | C4 Highly active (11.2%) | |
| --- | --- | --- | --- | --- | --- | --- | --- | --- |
|  | n | % or Mean (SD) | n | % or Mean (SD) | n | % or Mean (SD) | n | % or Mean (SD) |
| Sex | 3,040 |  | 8,355 |  | 8,809 |  | 2,545 |  |
| Male | 1,620 | 53.3 | 3,930 | 47.0 | 4,168 | 47.3 | 1,590 | 62.5 |
| Female | 1,420 | 46.7 | 4,425 | 53.0 | 4,641 | 52.7 | 955 | 37.5 |
| Age | 3,040 | 31.8 (9.4) | 8,355 | 30.2 (8.9) | 8,810 | 29.8 (8.8) | 2,545 | 27.9 (8.4) |
| Health status |  |  |  |  |  |  |  |  |
| Cardiovascular diseases ^a^ | 3,157 |  | 7,860 |  | 7,541 |  | 2,030 |  |
| No | 2,982 | 94.5 | 7,517 | 95.6 | 7,252 | 96.2 | 1,975 | 97.3 |
| Yes | 175 | 5.5 | 343 | 4.4 | 289 | 3.8 | 55 | 2.7 |
| Education | 2,980 | 7.5 (2.5) | 8,165 | 8.4 (3.1) | 8,593 | 8.8 (3.3) | 2,466 | 9.1 (3.4) |
| BMI | 2,549 | 24.3 (3.7) | 7,402 | 23.6 (3.4) | 7,856 | 23.3 (3.3) | 2,266 | 23.1 (2.9) |
| Smoking | 2,507 |  | 7,319 |  | 7,787 |  | 2,235 |  |
| Never | 1,069 | 42.6 | 3,208 | 43.8 | 3,580 | 46 | 1,075 | 48.1 |
| Occasional | 65 | 2.6 | 230 | 3.1 | 247 | 3.2 | 82 | 3.7 |
| Former | 457 | 18.2 | 1,515 | 20.7 | 1,683 | 21.6 | 493 | 22.1 |
| Light | 162 | 6.5 | 569 | 7.8 | 593 | 7.6 | 151 | 6.8 |
| Medium | 382 | 15.2 | 1,051 | 14.4 | 1,017 | 13.1 | 264 | 11.8 |
| Heavy | 372 | 14.8 | 746 | 10.2 | 667 | 8.6 | 170 | 7.6 |
| Alcohol use | 2,500 |  | 7,325 |  | 7,757 |  | 2,237 |  |
| Never | 323 | 12.9 | 643 | 8.8 | 632 | 8.1 | 170 | 7.6 |
| Former | 136 | 5.4 | 381 | 5.2 | 457 | 5.9 | 119 | 5.3 |
| Occasional | 156 | 6.2 | 394 | 6.4 | 388 | 5.0 | 107 | 4.8 |
| Low | 1,657 | 66.3 | 5,316 | 72.6 | 5,698 | 73.5 | 1,666 | 74.5 |
| Medium | 130 | 5.2 | 350 | 4.8 | 364 | 4.7 | 117 | 5.2 |
| High | 51 | 2.0 | 163 | 2.2 | 141 | 1.8 | 43 | 1.9 |
| Very high | 47 | 1.9 | 78 | 1.1 | 77 | 1.0 | 15 | 0.7 |
| BMI, body mass index; SD, standard deviation. ^a^ Self-reported physician-diagnosed angina pectoris or myocardial infarction. | | | | | | | | |


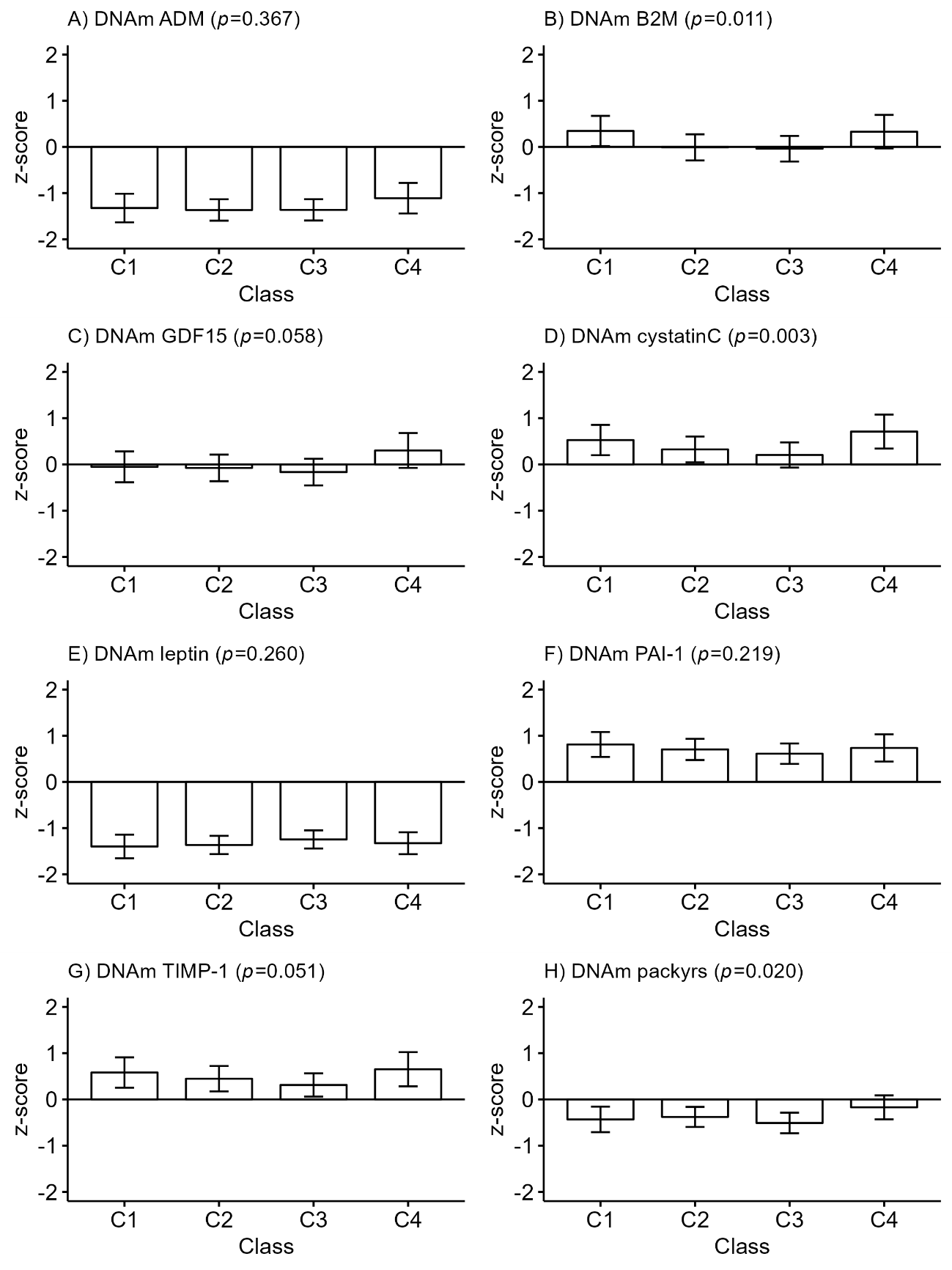


**Fig. S1** Mean differences in PC-based DNA methylation (DNAm)-based plasma proteins and smoking pack-years between the classes of long-term leisure-time physical activity (n = 1,153): A) DNAm adrenomedullin (ADM), B) DNAm beta-2 microglobulin (B2M), C) DNAm growth differentiation factor (GDF15), D) DNAm cystatin C, E) DNAm leptin, F) DNAm plasminogen activation inhibitor 1 (PAI-1), DNAm tissue inhibitor metalloproteinase 1 (TIMP-1), and H) DNAm smoking pack-years (packyrs). Means and 95% confidence intervals are presented. C1, Sedentary; C2, Moderately active; C3, Active; C4 Highly active. The model was adjusted for sex (female), age, timing of the blood drawn, education years, body mass index, smoking and alcohol use. P value from overall Wald test.


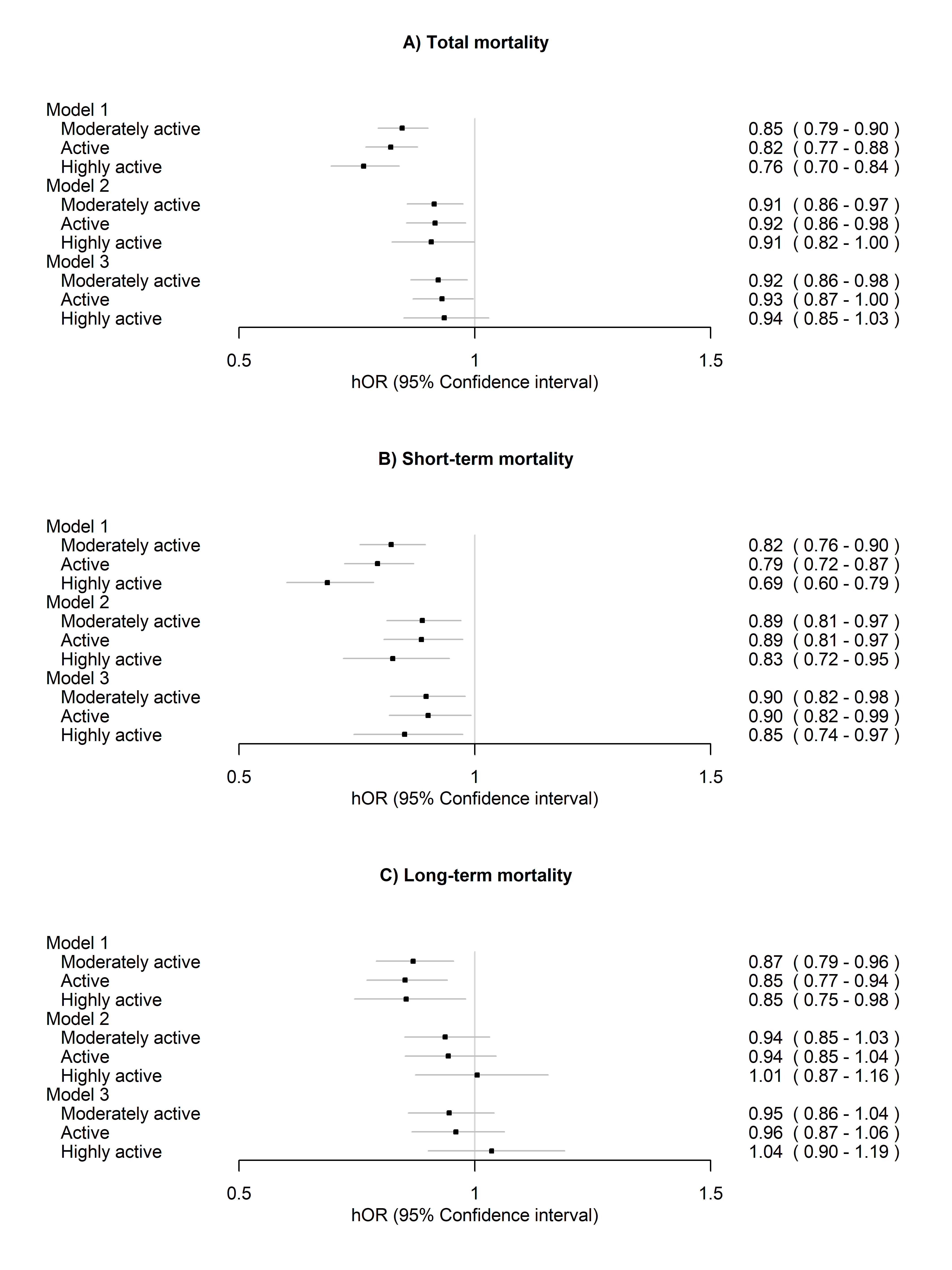


**Fig*.*** **S2** Associations of long-term leisure-time physical activity with A) total mortality, B) short-term mortality (1990–2011), and C) long-term mortality (2012–2020). Twins who did not report prevalent cardiovascular diseases (angina pectoris or myocardial infarction) were included in the analysis (n=19,726). Sedentary class was treated as reference.

Model 1 was adjusted for sex (female) and age*.* Model 2 was additionally adjusted for education, smoking and alcohol use and Model 3 for body mass index. hOR, hazard odds ratio.


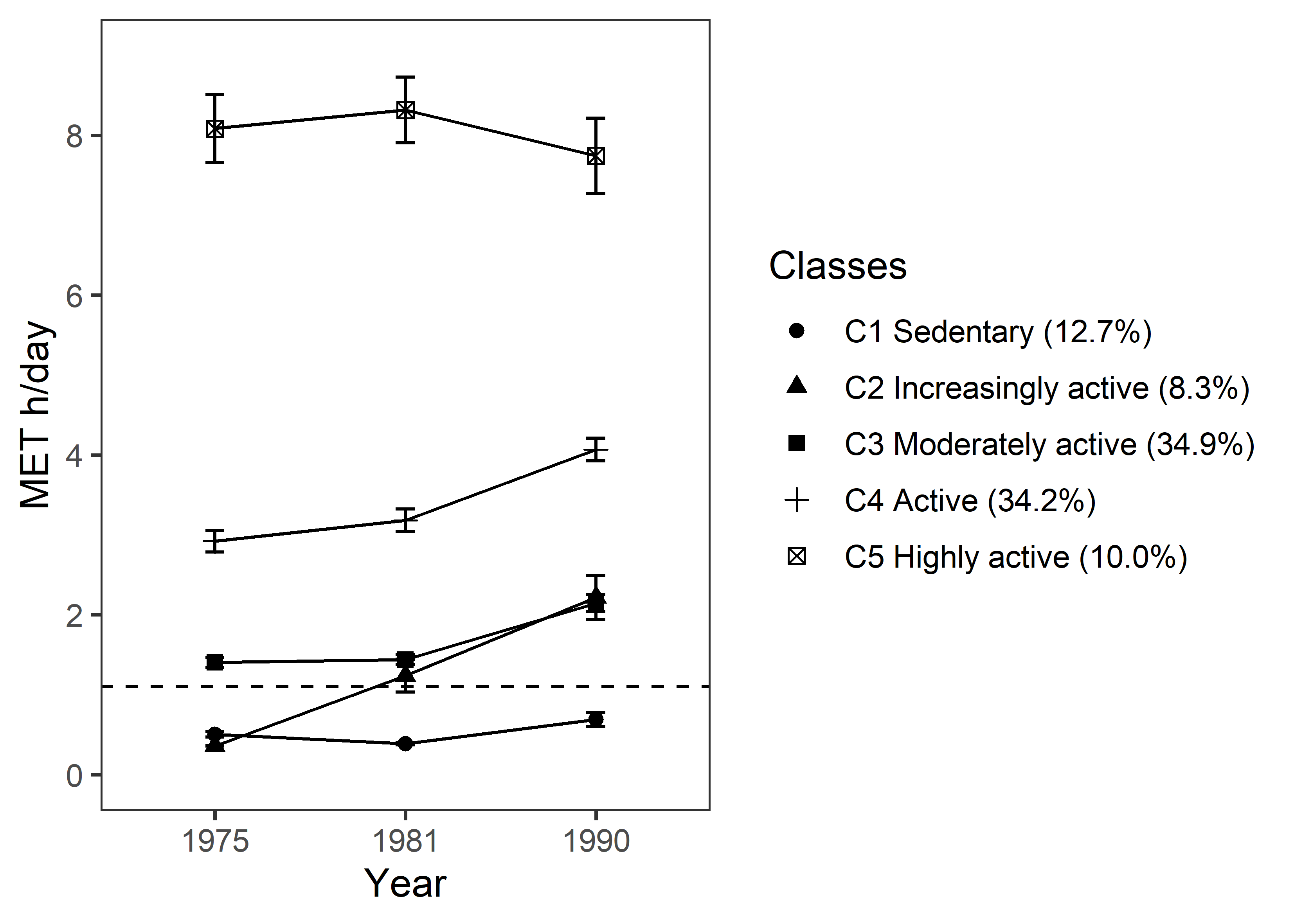


**Fig. S3** Sensitivity analysis: Latent profile solution with five classes (n = 22,750). Means of metabolic equivalent (MET) hours (h)/day and 95% confidence intervals are presented. The dashed line denotes World Health Organization guidelines for the recommended minimum amount of physical activity for adults (150 min of moderate intensity physical activity per week ~ 1.1 MET h/day).


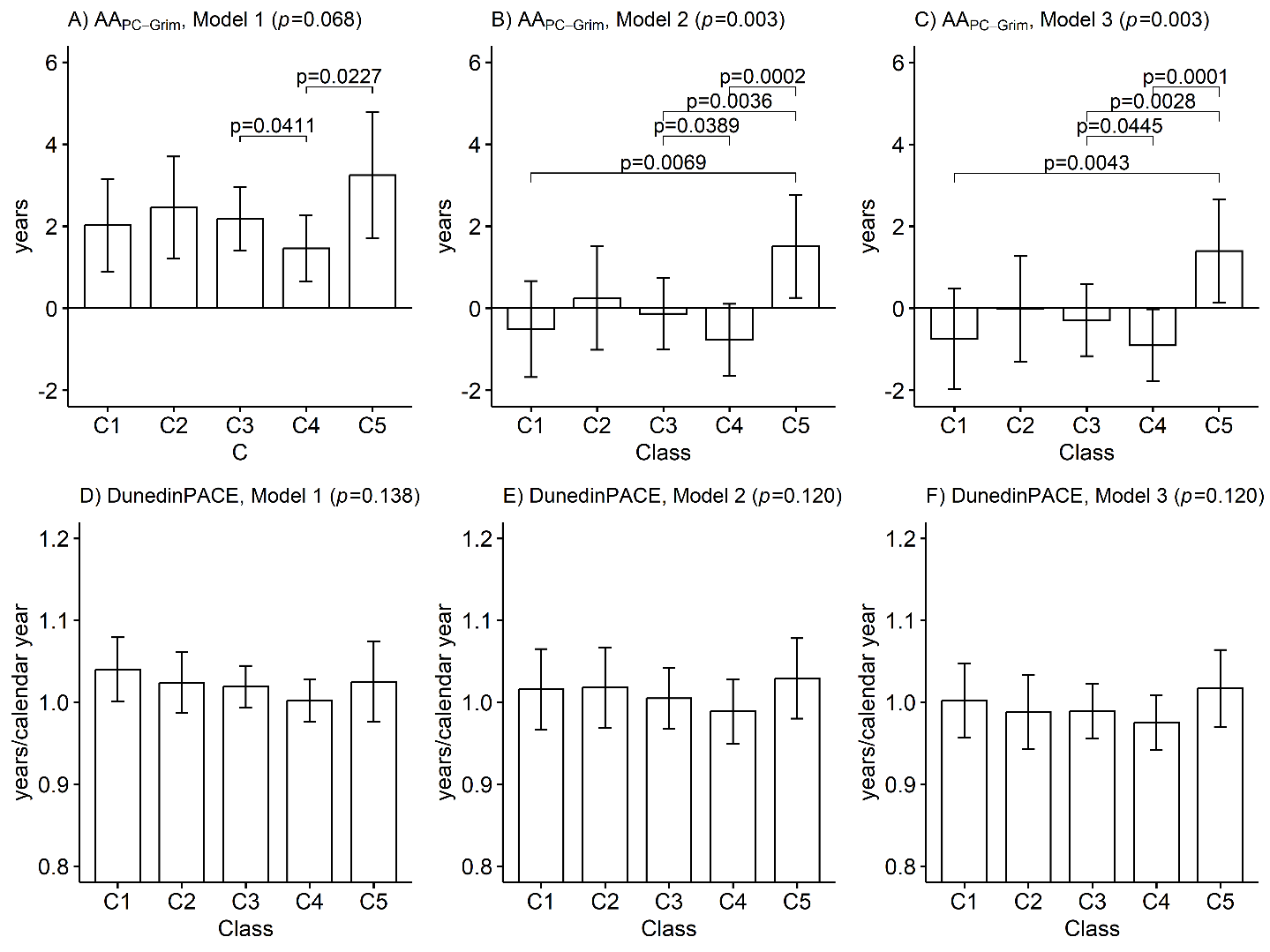


**Fig. S*4*** Sensitivity analysis: Latent profile solution with five classes; mean differences between the classes of long-term leisure-time physical activity in biological ageing measured with A–C) PC-based GrimAge and D–F) DunedinPACE (n=1,153). Means and 95% confidence intervals are presented. Model 1 was adjusted for sex (female), age and timing of the blood drawn. Model 2 was additionally adjusted for education, smoking and alcohol use and Model 3 additionally for body mass index. C1, Sedentary (8.0%); C2, Increasingly active (from sedentary to moderate) (7.7%); C3, Moderately active (39.3%); C4, Active (39.4%); C5 Highly active (5.6%); AA, Age acceleration. p value from Wald test.


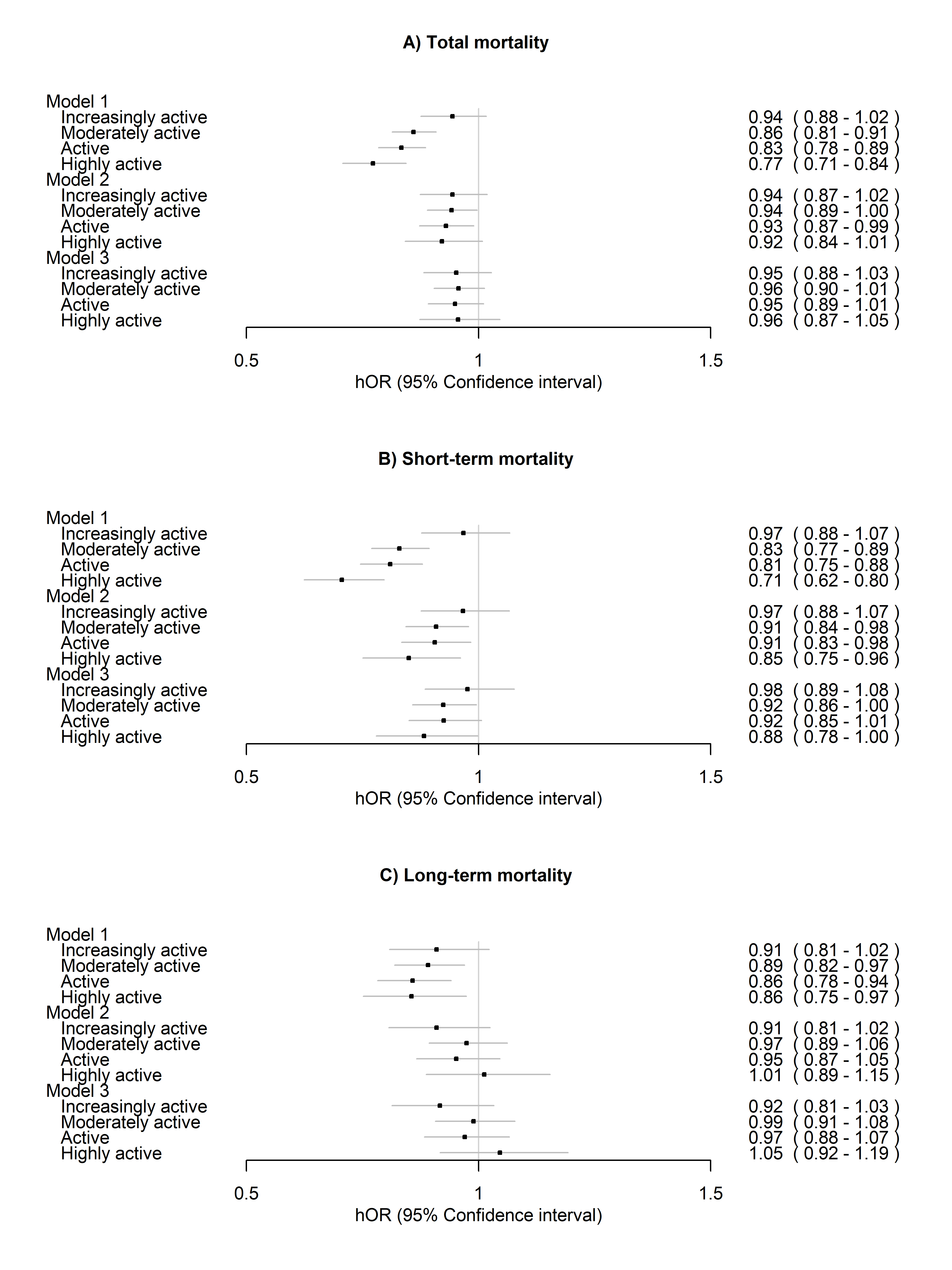


**Fig. S5** Sensitivity anaysis: Latent profile solution with five classes, the associations of long-term leisure-time physical activity with A) total mortality, B) short-term mortality (1990–2011), and C) long-term mortality (2012–2020) (n=22,750). Sedentary class was treated as reference. Model 1 was adjusted for sex (female) and age. Model 2 was additionally adjusted for education, smoking, and alcohol use and Model 3 additionally for body mass index.

hOR, hazard odds ratio.


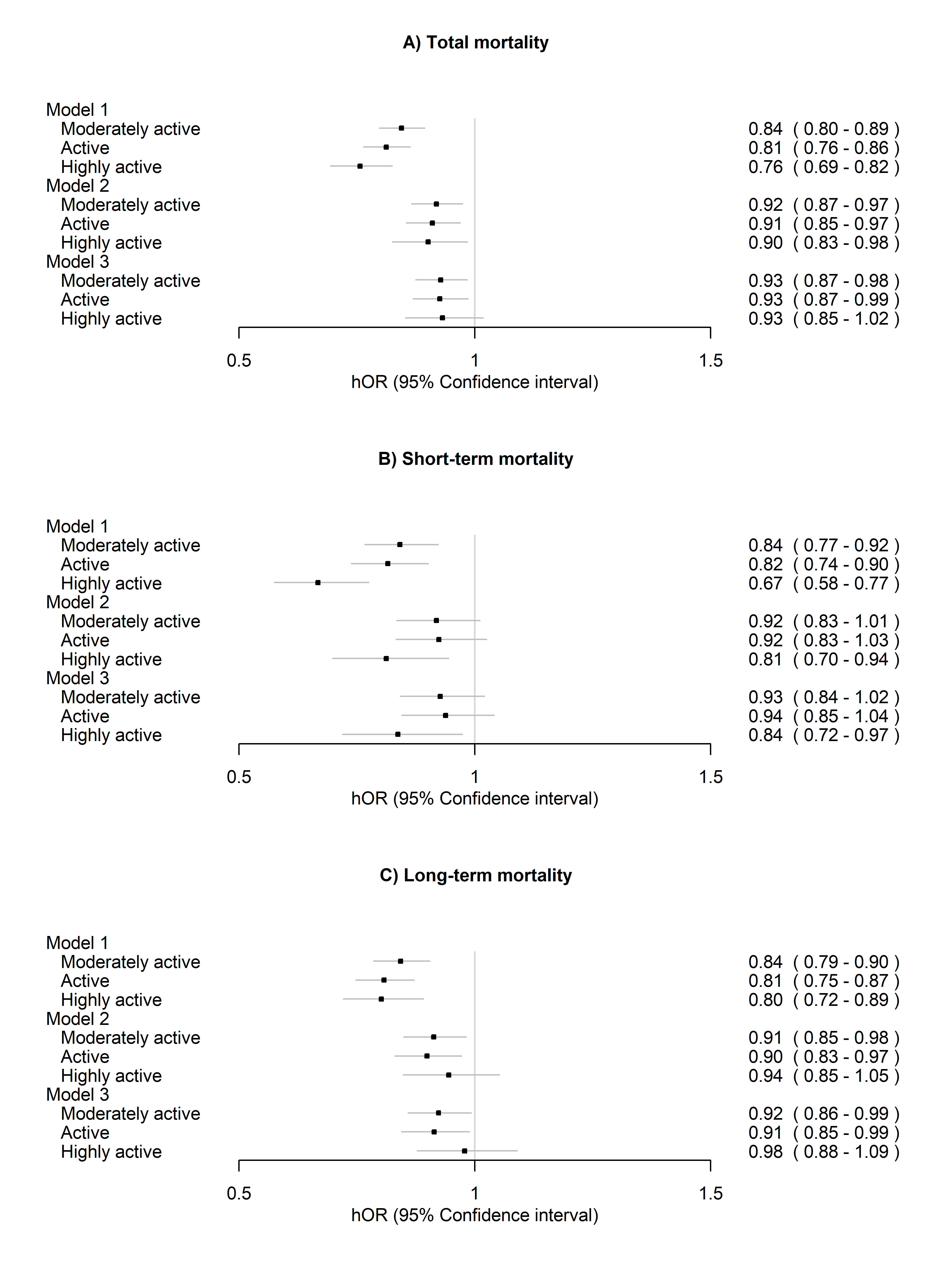


**Fig. S6** Sensitivity analysis: The associations of long-term leisure-time physical activity with A) total mortality, B) short-term mortality (1990–2006), and B) long-term mortality (2007–2020) (n = 22,750). Sedentary class was treated as reference. Model 1 was adjusted for sex (female) and age. Model 2 was additionally adjusted for education, smoking, and alcohol use and Model 3 additionally for body mass index. hOR, hazard odds ratio.
